# Dataset on the effect of knockout of KLK8 in social memory

**DOI:** 10.1016/j.dib.2019.104458

**Published:** 2019-08-30

**Authors:** Hitomi Nakazawa, Yuka Suzuki, Yasuyuki Ishikawa, Yoshio Bando, Shigetaka Yoshida, Sadao Shiosaka

**Affiliations:** a Department of Functional Anatomy and Neuroscience, Asahikawa Medical University, Asahikawa, Hokkaido, 078-8510, Japan; b Department of Systems Life Engineering, Maebashi Institute of Technology, Maebashi, Gunma, 371-0816, Japan; c Department of Anatomy, Akita University Graduate School of Medicine, Akita, Akita, 010-8543, Japan; d Emeritus Professor of Graduate School of Biological Science, Nara Institute of Science and Technology (NAIST), Ikoma, Nara, 630-0192, Japan

## Abstract

The data presented in this article have been produced as supporting data of the original research article titled “Impaired social discrimination behavior despite normal social approach by kallikrein-related peptidase 8 knockout mouse” (Nakazawa et al., 2019). Sociability and recognition of conspecifics and discrimination among conspecifics (social memory) is fundamental for pair bonding, to create social hierarchy, and eventually establish affiliated societies in social animals, including humans. It has been speculated that the processes of cognition, attention and memory, which are largely mediated by the hippocampus, contribute to social behavior. However, the molecular basis of social behavior remains elusive. This article presents a dataset of behavior-related KLK8-NRG1-ErbB signaling changes in the hippocampus and the effect of activation of ErbB signaling on social behavior.

**Table:** Specifications Table

Subject area	Biology
More specific subject area	Neurobiology, molecular and cellular neuroscience
Type of data	Figure, Graph, Image
How data was acquired	Microscope, Immunohistochemistry, Immunoblotting
Data format	Raw and analyzed data
Experimental factors	KLK8 knockout mice were used for experiments
Experimental features	Microscope, immunoblotting
Data source location	Department of Functional Anatomy and Neuroscience, Asahikawa Medical University, Asahikawa, Hokkaido, Japan
Data accessibility	Data are available with this article
Related research article	Nakazawa et al., “Impaired social discrimination behavior despite normal social approach by kallikrein-related peptidase 8 knockout mouse”, Neurobiol. Learn. Mem. 162, (2019) [1]

**Table:** 

**Value of the data** • Mouse's territory invasion by a novel mouse induced up-regulation of immediate early genes, KLK8 enzyme activity, and phosphorylation of ErbB4 (pErbB4), whereas mouse's territory invasion by a familiar mouse or a novel object did not induce up-regulation of KLK8 enzyme activity. These datasets are of value to researchers who need the information for studying behavior-related molecular signaling. • Intraventricularly injection of 0.5 nM NRG1177-246 induced up-regulation of pErbB4. Intraventricularly injection of JNJ-28871063 (JNJ) (10 mg/kg) inhibited up-regulation of pErbB4. These datasets are of value to researchers who need the information for studying the role of ErbB signaling *in vivo*. • In Crawley's three-chamber behavioral test, wild-type mice intraventricularly injected with NRG1177-246 impaired social memory. The data provided information that excessive activation of ErbB signaling impaired social memory. • To analyze the effect of drug treatment on social behavior, we performed intracerebroventricular cannula implantation to mice. The data can promote further research on signaling molecules controlling social behavior *in vivo*.

## 1 Data

**Fig. 1:**
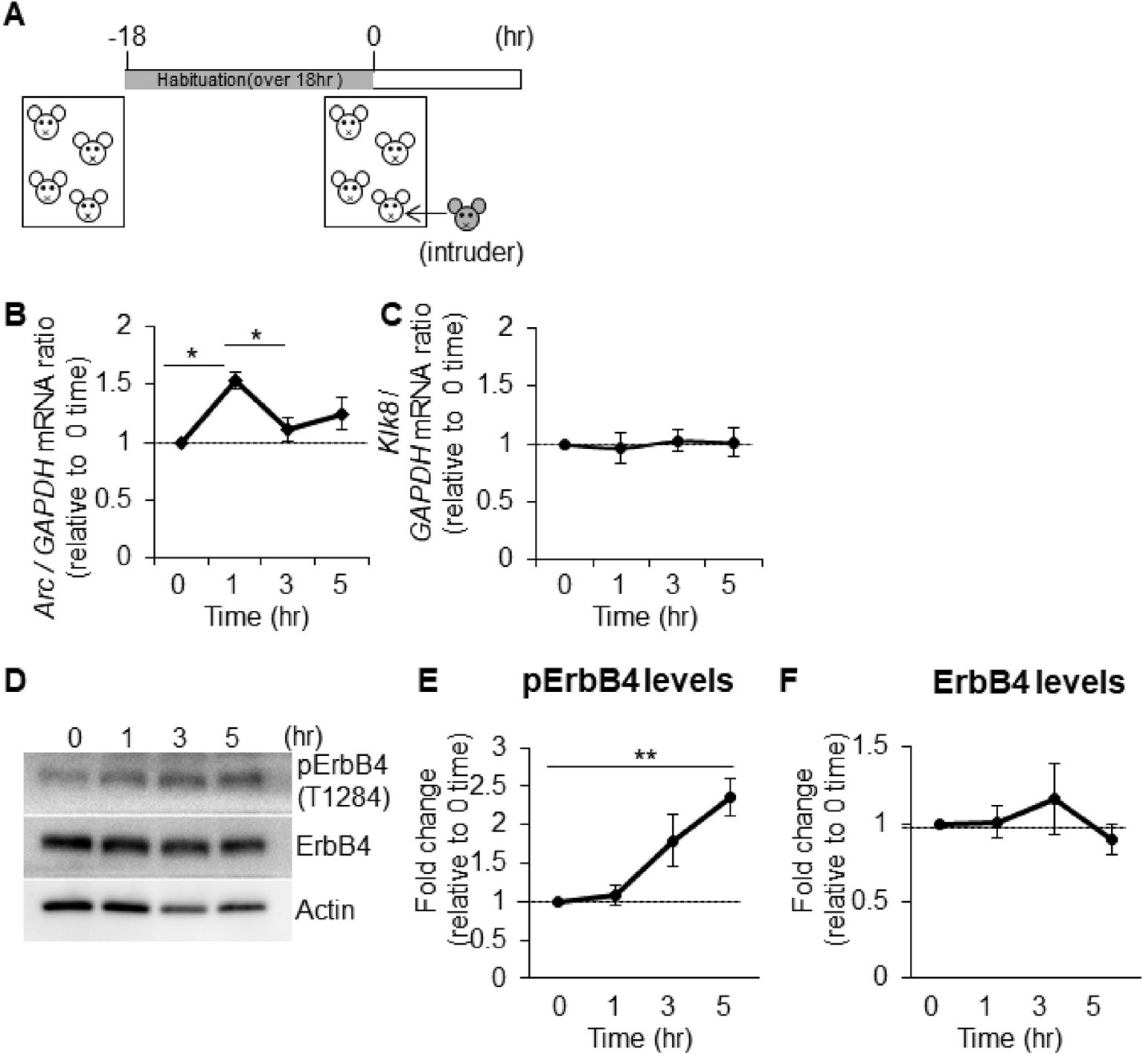
**Territorial invasion by a novel mouse induced several molecular changes in the recipient mouse hippocampus**. **A,** Schematic drawing of the mouse territory invasion paradigm. **B,** Quantitative analysis of *Arc* mRNA in the hippocampus after intrusion by a novel mouse (one-way ANOVA with post-hoc Tukey-Kramer, *p < 0.05, n = each 3). **C,** Quantitative analysis of *Klk8* mRNA. **D,** Representative western blots showing the levels of pErbB4, total ErbB4, and β-actin in the hippocampus of WT mice. **E and F,** Quantitative densitometric analysis of western blots. Ratios for pErbB4/ErbB4 (E) and ErbB4/β-actin (F) are shown. The fold change was normalized to the 0 h time-point (one-way ANOVA with post-hoc Tukey-Kramer, **p < 0.01, n = each 5); error bars indicate ± S.E.M. Dotted line in B, C, E, and F corresponds to a baseline.

**Fig. 2:**
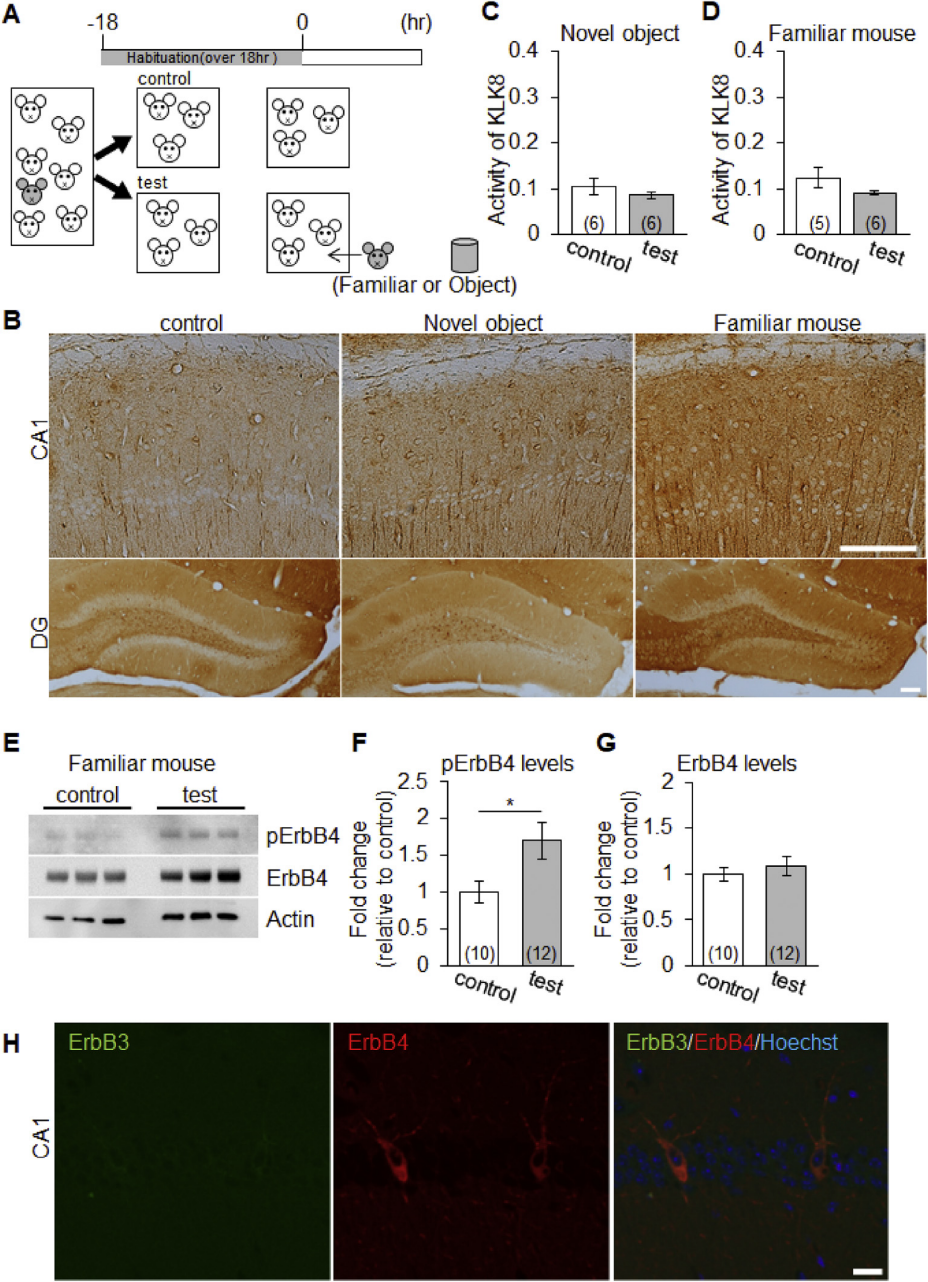
**Territorial invasion by a novel object or familiar mouse showed no significant increase in Arc immunoreactivity proteolytic activity of KLK8**. **A,** Schematic drawing of the mouse territory invasion paradigm. After habituation for 18 h, a familiar mouse and novel object (indicated in grey) was introduced into the mouse territory. **B,** Immunohistochemical analysis of Arc in the hippocampal CA1 and DG subfield 1 h after invasion by a novel object or familiar mouse. Scale bar, 100 μm. **C and D,** Average KLK8 activity ( × 10^−4^ U/μl, arbitrary units) at 3 h in control (white bar) and test mice (grey bar). **E,** Representative western blots showing the levels of phosphorylated ErbB4 (pErbB4), total ErbB4, and β-actin in the hippocampus from WT mice after invasion by a familiar mouse. **F and G,** Quantitative densitometric analysis of western blots. Ratios for pErbB4/ErbB4 (F) and ErbB4/β-actin (G) are shown. The fold change was normalized to the control group (Welch's t-test, *p < 0.05). Numbers inside the columns indicate the number of mice used. Error bars indicate ± S.E.M. **H,** Immunohistochemical analysis of ErbB3 and ErbB4 in the hippocampal CA1 subfield. Scale bar, 20 μm.

**Fig. 3:**
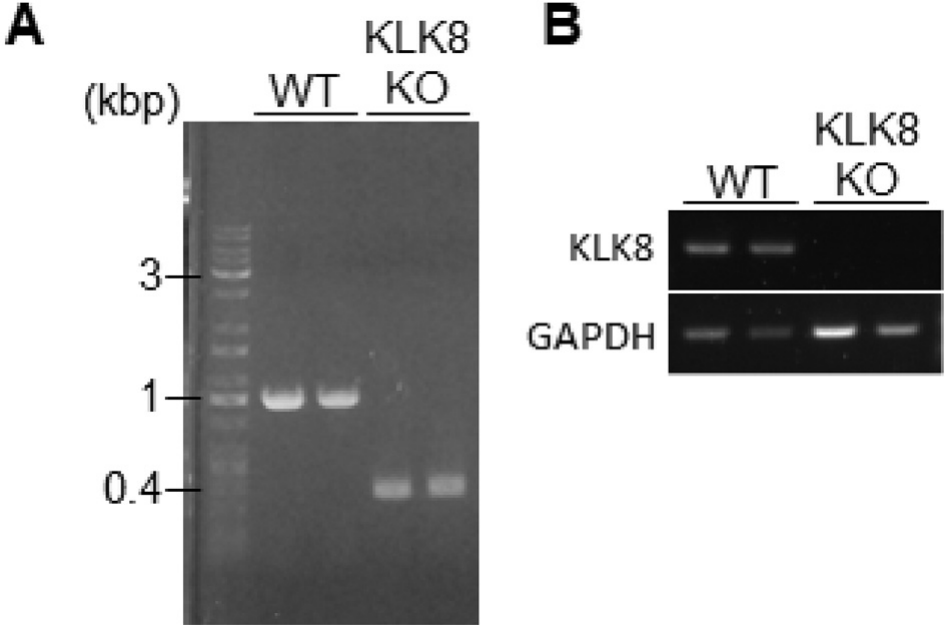
**Genotyping *Klk8*-KO and WT**. **A**, Genotyping *Klk8*-KO and WT mice by PCR. PCR on genomic DNA with the WT could produce a DNA fragment of 1,000 bp, and genomic DNA with *Klk*8-KO could produce a DNA fragment of 350 bp. **B**, Conﬁrmation of knockout of *Klk*8 DNA by reverse transcription PCR.

**Fig. 4:**
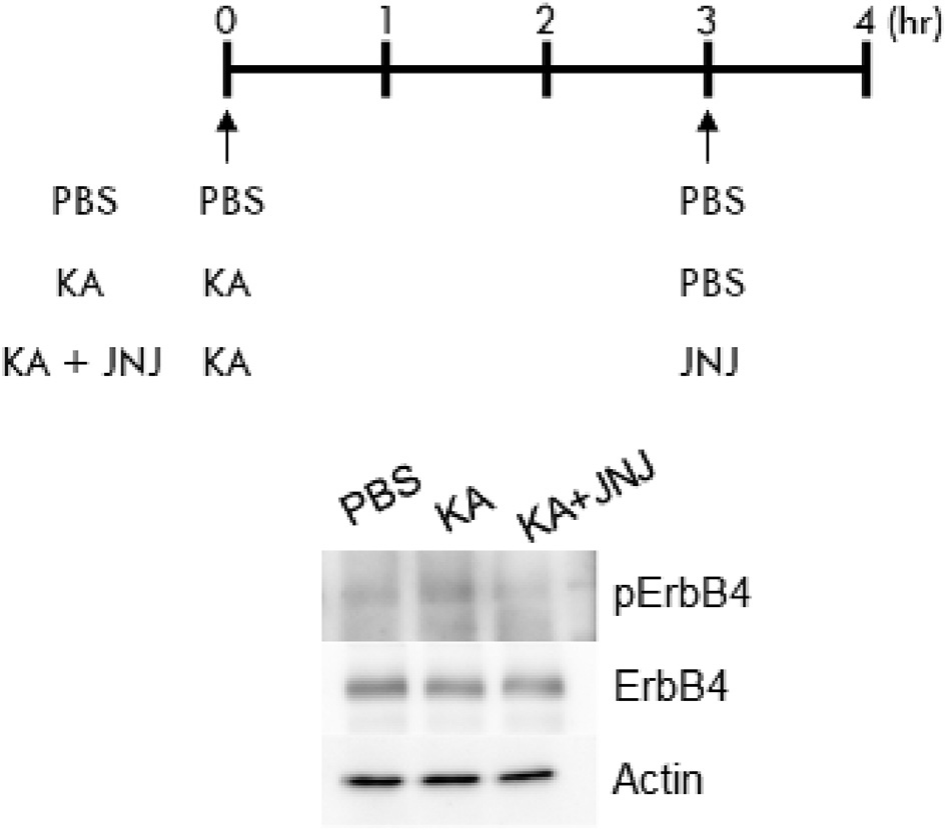
**The ErbB was blocked by JNJ-28871063**. Schematic drawing of JNJ-28871063 treatment paradigm. WT mice were injected with Kainate (KA; 25 mg/kg i. p.), Kainate acid and JNJ (10 mg/kg i. p.), or PBS. Representative western blots showing the levels of phosphorylated ErbB4 (pErbB4), total ErbB4, and β-actin in the hippocampus from drug-treated mice.

**Fig. 5:**
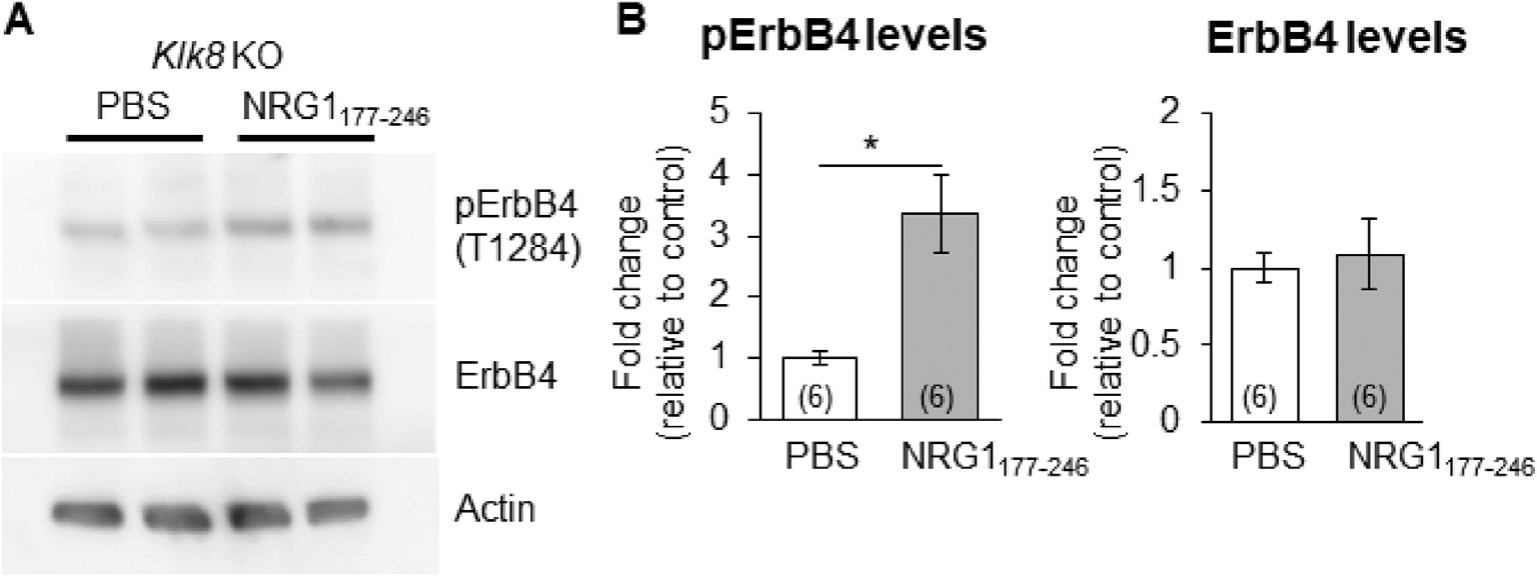
**Intraventricular injection with 0.5 nM NRG1_177-246_ induced up-regulation of pErbB4. A**, Representative western blots showing the levels of pErbB4, total ErbB4, and β-actin in the hippocampus from *Klk8* KO mice 1 h after injection of PBS or 0.5 nM NRG1_177-246_. **B**, Quantitative densitometric analysis of western blots. Ratios for pErbB4/ErbB4 and ErbB4/β-actin are shown. The fold change was normalized to the control group (PBS) (Welch's t-test, *p < 0.05). Numbers inside the columns indicate the number of mice used. Values represent the mean ± S.E.M.

**Fig. 6:**
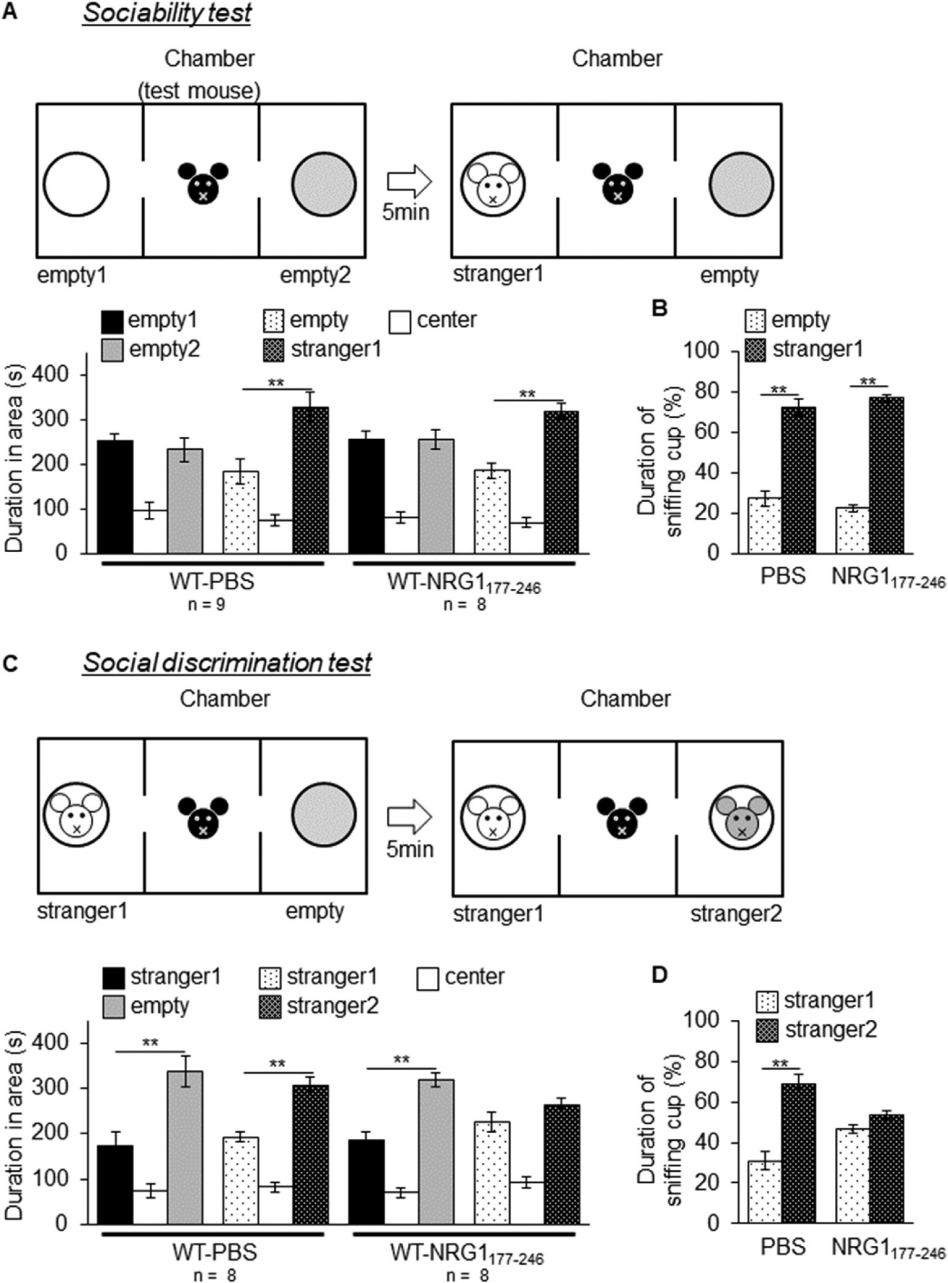
**Social discrimination was impaired in NRG1**_**177-246**_**-injected WT mice**. **A,** Schematic drawing of the sociability test. The graph shows results of the sociability test. Time spent in each chamber by PBS-injected and NRG1_177-246_-injected WT mice is shown (one-way ANOVA with post-hoc Tukey-Kramer, **p < 0.01). **B,** Time spent sniffing each chamber (%) is shown (one-way ANOVA with post-hoc Tukey-Kramer, **p < 0.01). **C,** Schematic drawing of the social discrimination test. The graph shows the social discrimination test results. Time spent in each chamber is shown (one-way ANOVA with post-hoc Tukey-Kramer, **p < 0.01). Values represent the mean ± S.E.M. **D**, Time spent sniffing each chamber (%) is shown (one-way ANOVA with post-hoc Tukey-Kramer, **p < 0.01). Numbers under the graph indicate the number of mice used.

The data give details of analyses of the behavior-related molecular changes in the territory invasion paradigm. Schematic in Fig. 1A is a drawing of the mouse territory invasion paradigm for temporal analysis. Quantitative real-time PCR analysis showed significant increase in *Arc* mRNA levels at 1 h after invasion of a novel mouse, but not in *Klk8* mRNA (Fig. 1B and C). The effect of the invasion of novel mouse on ErbB4 signaling was confirmed by immunoblotting with phosphorylated ErbB4 specific antibody (Fig. 1D). Quantification of the immunoblotting data showed increased phosphorylation of ErbB4 (pErbB4/ErbB4) at 5 h after invasion of novel mouse (Fig. 1E), and no significant changes of ErbB4 protein levels (ErbB4/β-actin, Fig. 1F). Fig. 2 shows the mouse territory invasion paradigm comparison of control group with test group invaded by a novel object or familiar mouse (Fig. 2A). Weak Arc immunoreactivity was seen in DG granular neurons of the hippocampus at 1 h after invasion of a novel object (a plastic cylinder) or familiar mouse (Fig. 2B). The protease activity of KLK8 ( × 10^−4^ U/μl, arbitrary units) showed no significant differences between control groups and test groups at 3 h after invasion of a novel object (Fig. 2C) or familiar mouse (Fig. 2D). In immunoblotting with an antibody specific for pErbB4, increased phosphorylation of ErbB4 was observed at 5 h after invasion of a familiar mouse (Fig. 2E). The phosphorylation of ErbB4 (Fig. 2F) were significantly increased after invasion of a familiar mouse, whereas there was no significant difference in the ErbB4 protein level (Fig. 2G). Immunohistochemical analysis with antibodies specific for ErbB3 and ErbB4 was showed that few protein expressions of ErbB3 in the hippocampal CA1 subfield (Fig. 2H). The genotype of *Klk*8-KO mice was conﬁrmed by PCR on genomic DNA (Fig. 3A) or mRNA (Fig. 3B). The effect of JNJ-28871063, a pan-ErbB kinase inhibitor, was conﬁrmed *in vivo*. When WT mice were intraventricularly injected with JNJ-28871063 (10 mg/kg), up-regulation of pErbB4 by kainate were inhibited (Fig. 4). The effect of NRG1_177-246_, a receptor tyrosine kinase ligand, was conﬁrmed *in vivo*. When *Klk8*-KO mice were intraventricularly injected with 0.5 nM NRG1_177-246_, hippocampal pErbB4 levels increased (Fig. 5A). The phosphorylation of ErbB4 (pErbB4/ErbB4, Fig. 5B) were significantly increased by injection of 0.5 nM NRG1_177-246_, whereas there was no significant difference in the ErbB4 protein level (ErbB4/β-actin, Fig. 5B; *p < 0.05, n = 6 for each mouse). We analyzed the impact of the intraventricular injection of NRG1_177-246_ on social behavior. To quantify social behavior, we performed the three-chamber test (Fig. 6). The time spent in the each chamber (Fig. 6A and C) and the time sniffing each wire cage (Fig. 6B and D) were measured. When both cages were empty, no significant preference between the two chambers was detected for either mice (Fig. 6A)**.** In the sociability test, both PBS-injected mice and NRG1_177-246_ injected mice showed a similar preference for the chamber with a cage containing stranger 1 (Fig. 6A and B; **p < 0.01, n = 9 for PBS-injected mice, n = 8 for NRG1_177-246_ injected mice**)**. In the social discrimination test (social memory), PBS-injected mice demonstrated normal sociability and social memory, whereas NRG1_177-246_-injected mice impaired social memory (Fig. 6C and D; **p < 0.01, n = 8 for PBS-injected mice, n = 8 for NRG1_177-246_ injected mice).

## 2 Experimental design, materials and methods

### 2.1 Biochemical and histological analyses

#### 2.1.1 Effect of territorial behavior on activity-related proteins, Klk8 and p-ErbB4

For analysis of hippocampal mRNA expression of immediate early genes and phosphorylated ErbB4 levels during mouse territory invasion, sibling group-housed male mice (4 male mice of 8–10 weeks of age indicated as white mice in Fig. 1A) were habituated in a novel cage for 18 h. Mice were left for 1, 3 and 5 h and then given a lethal dose of an anesthetic drug (0.3 mg/kg medetomin, Meiji Seika Pharma Co., Tokyo, Japan; 4 mg/kg midazolam, Fuji Pharma Co., Tokyo, Japan; 5 mg/kg butorphanol tartrate, Meiji Seika Pharma Co.). The hippocampal tissues were removed and used for immunohistochemistry, proteolytic activity and western blotting. Each value was normalized to the control groups. Values represent the mean ± S.E.M. of three to five independent experiments (Fig. 1).

#### 2.1.2 Effect of territorial behavior on activity-related proteins, Klk8 and pErbB4

Analysis of hippocampal protein expression of immediate early genes and proteolytic activity of KLK8 during mouse territory invasion can be considered as molecular changes of social behavior. Before habituation in a novel cage for 18 h, sibling group-housed male mice (5−6 male mice of 8–10 weeks of age indicated as white mice in Fig. 2A) were divided into two groups (test and control group). A familiar mouse and novel object mouse (indicated in grey in Fig. 2A) was introduced into the test group territory. Test and control group mice were left for 1, 3 and 5 h and then given a lethal dose of an anesthetic drug. The hippocampal tissues were removed and used for immunohistochemistry, proteolytic activity and western blotting. Each value was normalized to the control groups. Values represent the mean ± S.E.M (Fig. 2).

#### 2.1.3 Immunohistochemical analysis of hippocampal tissue

Mice deeply anesthetized with mixed anesthetic were transcardially perfused with saline followed by 4% paraformaldehyde in PBS. Brains were removed, postfixed overnight in the same fixative at 4 °C, rinsed twice with PBS, and placed in 30% sucrose in PBS for 2 d at 4 °C. The brains were then frozen quickly and cut into serial coronal sections (30 μm thickness) on a cryostat. Sections were collected as free-floating sections in PBS, incubated in blocking solution (5% BSA and 0.3% Triton X-100) for 1 h at room temperature and then incubated overnight at 4 °C with the following antibodies: rabbit anti-Arc (1:3,000; synaptic systems, Goettingen, Germany), anti-ErbB4 (Ab77) (H4.77.16; 1:200, Novus Biologicals, Colorado, U.S.A.), and rabbit monoclonal anti-ErbB3 (D22C5, 1:200, Cell Signaling Technology, Massachusetts, U.S.A.). Sections were then washed and incubated overnight at 4 °C with biotinylated anti-Rabbit IgG secondary antibodies (1:1,000; Vector laboratories, California, U.S.A.). Histology images were acquired using a Digital microscope (Nikon, Tokyo, Japan) equipped with Plan Apo 20 × , 0.75 NA and 10 × , 0.45 NA and Plan Fluor 4 × , 0.13 NA objectives, and a Digital sight ds-5m (Nikon). For immunohistochemical analysis with ErbB, sections were incubated with either Alexa-488-conjugated or Alexa-594-conjugated secondary antibodies (1:1000, Thermo Fisher Scientific, Massachusetts, U.S.A.) followed by nuclear staining with Hoechst 33342 dye (Dojindo Laboratories, Kumamoto, Japan). Sections were then imaged using a confocal laser scanning microscope (Olympus, Tokyo, Japan), and images were analyzed using FV1000-D image analysis software (Olympus) (Fig. 2H).

#### 2.1.4 Measurement of KLK8 enzyme activity

Measurement of the proteolytic activity of KLK8 was conducted as described previously [2]. Briefly, mice were euthanized by decapitation and hippocampal tissue from each mouse was homogenized in 0.75 ml lysis buffer (50 mM HEPES, pH 7.5, 150 mM NaCl, 5 mM EDTA, 1% Triton X-100 and 10 μg/ml leupeptin) and then centrifuged at 15,000×*g* for 30 min at 4 °C to remove debris. The supernatant was incubated with the rat monoclonal anti-mouse KLK8 antibody mAbF12 (0.1 μg; Molecular and Biological Laboratories, Nagoya, Japan) overnight at 4 °C. The immunoprecipitated KLK8 was incubated with the synthetic fluorescent substrate, Pro-Phe-Arg-4-methyl-coumaryl-7-amide (Peptide Institute, Osaka, Japan) at 37 °C for 18 h. The product (7-amino-4-methylcoumarin) was quantified using a fluorescence spectrophotometer (F-4500; Hitachi, Tokyo, Japan). KLK8 protease activity ( × 10^−4^ U/μl, arbitrary units) was calculated from a standard curve that was generated by serially diluting aliquots of recombinant KLK8 (Fig. 2C and D).

### 2.2 Western blot analysis

#### 2.2.1 Measurement of phospho-ErbB4 and total ErbB4

Mice were euthanized by decapitation, and the hippocampal region was rapidly dissected. The tissue was immersed in ice-cold isotonic sucrose buffer (50 mM HEPES, pH 7.5, 0.32 M sucrose, 5 mM EDTA, 1 mM sodium fluoride, 1 mM sodium orthovanadate, and 1% protease inhibitor cocktail) and homogenized on ice in a glass-Teflon homogenizer. Subsequent procedures were performed at 4 °C. Homogenates were centrifuged at 1,000×*g* for 5 min to remove nuclei. The supernatants were centrifuged again at 10,000×*g* for 20 min to pelletize the crude membrane fraction. The obtained membrane preparations were lysed in lysis buffer (5 mM HEPES, pH 7.5, 150 mM NaCl, 5 mM EDTA, 1% SDS, 1 mM sodium fluoride, 1 mM sodium orthovanadate, and 1% protease inhibitor cocktail), and the lysates were centrifuged at 14,000×*g* to remove debris. The protein concentrations of the supernatants were determined with a BCA protein assay kit (Thermo Fisher Scientific), and equal amounts of total protein were separated on 7.5% SDS-PAGE gels. Following transfer to nitrocellulose membranes (Bio-Rad Laboratories, California, U.S.A.), free protein-binding sites were blocked with 5% skimmed milk prepared in Tris-buffered saline containing 0.1% Tween-20 for 30 min. The membranes were incubated with rabbit monoclonal anti-phospho-ErbB4 (Y1284) (21A9, 1:1,000, Cell Signaling Technology, Massachusetts, U.S.A.) and HRP-conjugated mouse monoclonal anti-β-actin (ab20272, 1:40,000, Abcam, Cambridge, UK) antibodies overnight at 4 °C. Subsequently, the blots were developed using an HRP-conjugated donkey anti-rabbit IgG antibody (1:10,000, Jackson ImmunoResearch Laboratories, Pennsylvania, U.S.A.) and a chemiluminescent reagent (Immobilon Western, Millipore, Massachusetts, USA), and exposed to X-ray film (Fujifilm, Tokyo, Japan). The blots were stripped and hybridized with a rabbit polyclonal anti-C-terminal ErbB4 antibody (C-18) (sc-283, 1:600, Santa Cruz Biotechnology) to determine the total amount of ErbB4. We chose the quantified band at approximately 180 kDa. A rabbit polyclonal anti-C-terminal ErbB4 antibody (total ErbB4 antibody, sc-283) also detects a band at approximately 180 kDa. Band densities were quantified with ImageJ software. The staining of β-actin was used as a standard for protein quantification. The ratios of pErbB4/ErbB4 denoted by pErbB4 levels and the ratios of ErbB4/β-actin denoted by ErbB4 levels. Values represent the mean ± S.E.M.

### 2.3 PCR

#### 2.3.1 Genotype PCR

DNA from mice tails was extracted using genotype lysis buffer (50 mM Tris-HCl, pH 8.0, 200 mM NaCl, 25 mM EDTA, 0.1% sodium dodecyl sulfate, 100 μg/ml proteinase K). PCR was performed using the 3 following conditions: 94 °C for 40 s, 58 °C for 40 s, and 72 °C for 1 min. Primer sequences used for PCR were as follows: wild-type sense: 5′–GCCTTTCCTGACCACTCTAA-3′ and antisense: 5′-GCACCGTGACCTCTTCAAA-3′ for the wild-type allele, and neo sense:5′–GGCTTCTGAGGCGGAAAGAA- 3′ and antisense: 5′-GCACCGTGACCTCTTCAAA-3′ for the mutated allele (Fig. 3A).

#### 2.3.2 Quantitative real-time PCR

Total RNA was extracted from hippocampi using TRIzol reagent (Life Technologies, California, U.S.A.) according to the manufacturer's instructions. For cDNA synthesis, total RNA (0.5 μg) was reverse transcribed with TaqMan reverse transcription reagents using random hexamers (Life Technologies). Quantitative real-time PCR was conducted using a LightCycler Instrument (Roche Diagnostics, Basel, Switzerland) with SYBR Green PCR Master Mix (Roche Applied Science, Upper Bavaria, Germany). Following denaturation for 10 min at 95 °C, the reactions were cycled 40 times with denaturation at 95 °C for 10 s, annealing at 60 °C for 10 s and elongation at 72 °C for 14 s. The following primers were used to amplify specific cDNA regions of interest: *Arc* (sense, 5′-TGGGCACCCTGCAGCCCAAAC-3’; antisense, 5′-CTATTCAGGCTGGGTCCTGTCAC-3′), *Klk8* (sense, 5′-CCCACTGCAAAAAACAGAAG-3’; antisense, 5′-TGTCAGCTCCATTGCTGCT-3′) and *Gapdh* (sense, 5′-CGGGAAGCCCATCACCATC-3’; antisense, 5′-GAGGGGCCATCCACAGTCTT-3′) (Fig. 1B and C).

### 2.4 Behavioral analyses

#### 2.4.1 Sociability behavior test

The sociability behavior test was performed at 355 lux using male mice following the procedure described by Kaidanovich-Beilin [3], [4]. Three chambers – left (L), center (C), and right (R) (30 × 60 × 35 cm) were connected and the test mouse (indicated in black) could freely go to chamber L and chamber R from chamber C. A small cylindrical wire cage (10 cm in diameter) was put in each of chambers L and R. The test mice were habituated to the apparatus for 7 d before the test (15 min/d). On the test day, the test mice were habituated to the apparatus containing the two small cylindrical wire cages for 10 min. After the habituation period, the test mouse was enclosed in the center compartment, and a stranger mouse from another family (WT male, stranger 1) was placed into one of the two wire cages. The test mouse was allowed to explore the test chamber for 10 min. During the trial, mouse activity was recorded on video and the time spent in the chamber containing each wire cage and the time sniffing each wire cage were measured. Sociability is defined as the subject mouse spending more time in the chamber containing the stranger mouse than in the empty chamber. The location for stranger 1 alternated between the left and right sides of the social test box for consecutive test mice (Fig. 6).

#### 2.4.2 Social discrimination behavior test

In the social discrimination behavior test, discrimination behavior was analyzed at 355 lux using male mice following a previously described procedure [3], [4]. The time spent in the each chamber containing each wire cage and the time sniffing each wire cage were measured. We confirmed the sociability behavior and subsequently performed the social discrimination behavior test. The mice showing normal sociability determined by the sociability test were used in the social discrimination test. At the end of the 10-min sociability behavior test, the test mouse was enclosed in the center compartment of the three chambers, and another stranger mouse (stranger 2, WT male) was placed in the empty wire cage. The test mouse was then allowed to explore the chamber for 10 min [social discrimination test (social memory)]. The test mouse had a choice between the first, already investigated mouse (stranger 1) and the novel unfamiliar mouse (stranger 2). C57BL/6J mice show a preference for social novelty. Therefore, social discrimination behavior is defined as the subject mouse spending more time in the chamber containing the new stranger 2 than in the chamber containing the now-familiar stranger 1.

#### 2.4.3 Intracerebroventricular cannula implantation

Mice destined for intracerebroventricular treatment underwent surgical implantation of an intracerebroventricular cannula 2 weeks prior to behavioral experiments. Mice were anaesthetized with 2-3% isoflurane (AbbVie, Illinois, U.S.A.). Using a stereotaxic apparatus (Narishige, Tokyo, Japan), a 26 G double guide cannula was implanted into the brain, targeting the bilateral ventricle (−0.5 mm anteroposterior, ±1.0 mm mediolateral, and −2.0 mm dorsoventral relative to the bregma). The guide cannula was fixed to the skull with anchor screws (1.2 mm, Misumi, Tokyo, Japan) and super cement (Shofu, Kyoto, Japan). A dummy cannula was inserted into the guide to keep it clean and prevent occlusion. Following surgery, mice were group-housed and their recovery was monitored for 1 week. After recovery from surgery for 1 week, the mice were habituated to the three-chamber test apparatus for 7 d (15 min/d).

#### 2.4.4 Drug treatment

2 μl of 0.5 nM recombinant NRG1_177-246_ [human NRG1-β1/HRG1-β1 EGF domain CF, R&D Systems, Minnesota, U.S.A.; human NRG1_177-246_ shares 98.6% (100%) sequence identity (similarity) with the mouse NRG1_177-246_] was injected into the cerebral ventricle for 10 min. After injection, the mice were placed in the three-chamber apparatus for 20 min for habituation, and the behavioral test was subsequently performed.
